# Nanoscale volume confinement and fluorescence enhancement with double nanohole aperture

**DOI:** 10.1038/srep15852

**Published:** 2015-10-29

**Authors:** Raju Regmi, Ahmed A. Al Balushi, Hervé Rigneault, Reuven Gordon, Jérôme Wenger

**Affiliations:** 1 CNRS, Aix Marseille Université, Centrale Marseille, Institut Fresnel, 13013 Marseille, France; 2 Department of Electrical Engineering, University of Victoria, Victoria, British Columbia V8W 3P6, Canada

## Abstract

Diffraction ultimately limits the fluorescence collected from a single molecule, and sets an upper limit to the maximum concentration to isolate a single molecule in the detection volume. To overcome these limitations, we introduce here the use of a double nanohole structure with 25 nm gap, and report enhanced detection of single fluorescent molecules in concentrated solutions exceeding 20 micromolar. The nanometer gap concentrates the light into an apex volume down to 70 zeptoliter (10^−21^ L), 7000-fold below the diffraction-limited confocal volume. Using fluorescence correlation spectroscopy and time-correlated photon counting, we measure fluorescence enhancement up to 100-fold, together with local density of optical states (LDOS) enhancement of 30-fold. The distinctive features of double nanoholes combining high local field enhancement, efficient background screening and relative nanofabrication simplicity offer new strategies for real time investigation of biochemical events with single molecule resolution at high concentrations.

Plasmonic nanoantennas realize a new paradigm to concentrate light energy into nanoscale dimensions^1^, enhance the luminescence of quantum emitters^2^,^3^ and trap single nano-objects^4^. To overcome the diffraction limit, nanoantenna designs take advantage of sharp curvature radii, nanoscale gaps and plasmonic resonances, using metal nanoparticles^5^,^6^,^7^, nanorods^8^,^9^, dimer gap antennas^10^,^11^,^12^,^13^,^14^ or bowtie antennas^15^,^16^.

The enhanced detection of single fluorescent molecules in concentrated solutions is an emerging field of application for plasmonic antennas^17^,^18^. Transient interactions between proteins, nucleic acids, and enzymes typically occur at micromolar concentrations^19^,^20^, however single-molecule diffraction-limited confocal techniques are restricted to concentrations in the pico to nanomolar range due to femtoliter detection volumes. Reaching single molecule sensitivity at the physiologically relevant micromolar concentrations thus requires over three orders of magnitude reduction in the detection volume^21^,^22^. Sub-wavelength nanoapertures milled into optically thick metal layers (also called “zero-mode waveguides”) concentrate light at the bottom of the apertures^23^, constraining the detection volume to the attoliter range^24^,^25^,^26^. However, the fluorescence enhancement with single circular nanoapertures is limited around typically ten-fold and the signal-to-noise ratio rapidly deteriorates when the aperture diameter goes below 100 nm^27^. To get higher volume confinement and fluorescence enhancement factors, we have recently developed the “antenna-in-box” design^28^ combining a resonant gap antenna into a rectangular nanoaperture. Experimentally, this design provides fluorescence enhancement up to several hundred folds while the detection volume is confined in the range 70–100 zeptoliter (1 zL = 10^−21^ L). However, the fabrication of this structure remains quite demanding due to the complex shape of the antenna. Moreover, even for 80 nm particle antenna-in-box, the resonances are typically in the range 700–800 nm, away from the emission bands of the most common fluorescent dyes.

As alternative plasmonic antenna design, the double nanohole (DNH) structure milled into a metal film has recently attracted much interest to realize an efficient platform to trap single nano-objects^29^,^30^,^31^ down to the ultimate single protein level^32^,^33^,^34^,^35^. The DNH design has a distinctive set of advantages: (i) the apex between the two holes directly realizes sharp radii of curvatures and nanometer gap sizes, providing high local intensity enhancement, (ii) the optically thick metal film efficiently screens out the background from the solution around the structure, (iii) heating effects are avoided thanks to the good thermal conductivity of the gold film, and (iv) the structure remains relatively simple to fabricate as compared to bowtie antenna^16^,^36^, bowtie aperture^37^,^38^ or antenna-in-box^28^. These specific features make DNH highly relevant to enhance the detection of fluorescent molecules in concentrated solutions. Moreover, the quantification of the fluorescence enhancement factor in DNH is interesting for plasmon-enhanced luminescence applications, and the measurement of the apex near-field volume is important to better understand the phenomenon leading to enhanced plasmonic trapping in DNH.

Here we use double nanohole structure with 25 nm gap to enhance the detection of single fluorescent molecules in solutions up to 20 *μ*M concentration (Fig. 1a). Using fluorescence correlation spectroscopy (FCS), we measure the near-field apex volume to 70 zeptoliter, realizing a volume reduction of 7000-fold as compared to diffraction-limited confocal setups. This high intensity confinement goes with fluorescence enhancement up to 100-fold, together with microsecond transit time and single molecule sensitivity at concentrations exceeding 20 micromolar. We also conclusively demonstrate the acceleration of the fluorescence photodynamics in the nanometer apex region, and report experimentally 30-fold enhancement of the local density of optical states (LDOS), in good agreement with numerical simulations. In all the experiments, the polarization-dependent response ensures that the relevant signal stems from the nanoscale apex area, and not from the surrounding holes. The cutting-edge optical performance, the relative ease of nanofabrication and the efficient background screening make the DNH structure ideal to study complex biochemical dynamics at physiological concentrations.

## Results

### Zeptoliter volume with 100-fold fluorescence enhancement

**Figure 1:**
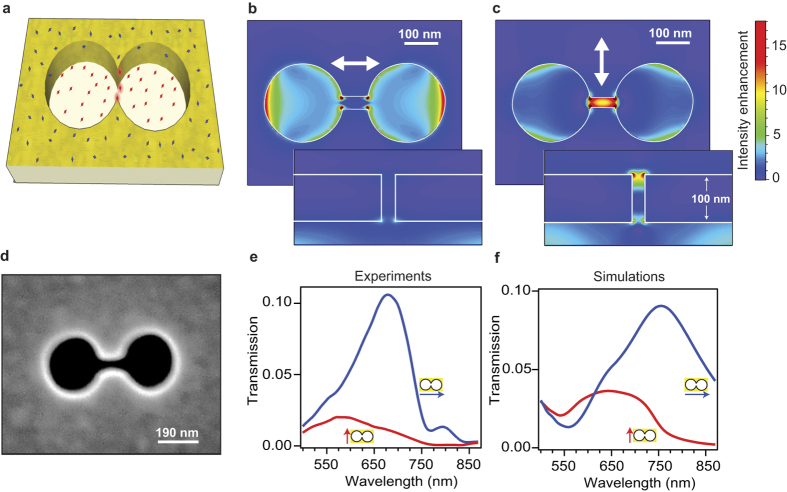
(**a**) Sketch of double nanohole (DNH) structure to enhance single molecule fluorescence in the apex region. (**b**,**c**) Local intensity enhancement (linear scale) for a DNH of 25 nm gap and 190 nm diameter excited at 633 nm with a linear polarization perpendicular (**b**) and parallel (**c**) to the apex between the holes, taken in a plane 5 nm below the top metal surface. The inserts show the intensity enhancement along a vertical cut in the DNH center. All images share the same colorscale. (**d**) Scanning electron microscope image of the structure milled in 100 nm thick gold film using focused ion beam. (**e**) Experimental and (**f**) simulated transmission spectra for a DNH illuminated with normal incidence for two orthogonal linear polarizations along the apex (red) and perpendicular to the apex (blue).

The DNH structure is realized by focused ion beam milling of two 190 nm diameter nanoholes connected by an apex region of 60 nm length and 25 nm gap width (Fig. 1d). The DNH bears a clear polarization dependence (Fig. 1b,c): when the incoming light polarization is oriented parallel to the apex between the nanoholes, light is mainly concentrated in the gap region. Conversely, when the incoming light polarization is oriented perpendicular to the apex, there is a minimum intensity in the gap as light is mainly concentrated in the nanohole region. This behavior is confirmed by recording far-field transmission spectra using polarized illumination (Fig. 1e,f). When the orientation is set parallel to the apex, a minimum of transmission is found as the electromagnetic intensity is concentrated in the gap, whereas a maximum transmission is obtained for a polarization oriented perpendicular to the apex^29^. These findings are well reproduced by FDTD numerical simulations of the transmission spectra (Fig. 1f). Moreover, the recorded spectra show that the DNH response covers well the 633 nm laser excitation wavelength and the 650–690 nm fluorescence emission band for the Alexa Fluor 647 dye.

**Figure 2:**
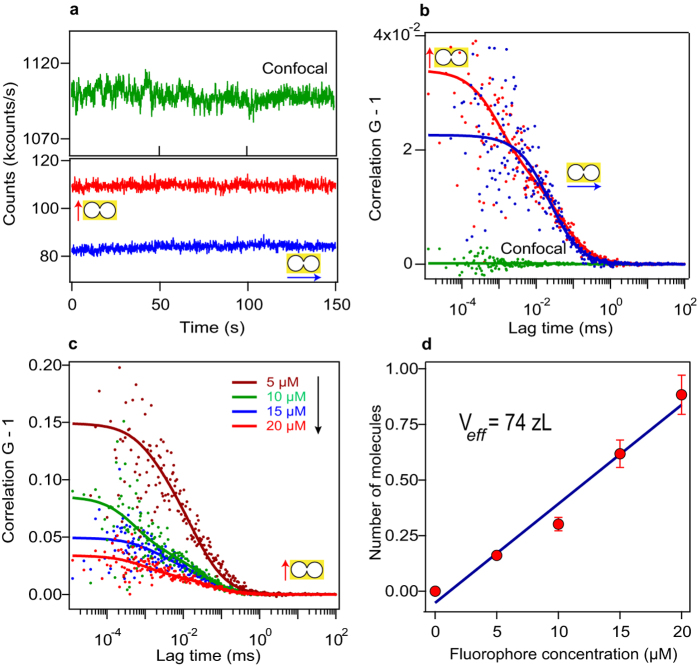
FCS analysis to measure the near-field apex volume. (**a**) Fluorescence time trace with excitation light parallel (red line) and perpendicular (blue line) to the apex region. The time trace found for the confocal case (0.5 fL diffraction-limited volume) is shown in green for comparison. (**b**) FCS correlation function of the traces shown in (**a**). For all cases, the Alexa Fluor 647 concentration 20 *μ*M with 200 mM of methylviologen as chemical quencher, and the excitation power is 10 *μ*W. Dots are experimental points, lines are fits using the model described in the Methods section. A higher correlation amplitude is observed with the polarization parallel to the apex, and corresponds to a lower number of detected molecule (stronger confinement of light). The fit parameters are summarized in Table 1. (**c**) FCS correlation functions for increasing concentrations of fluorescent dyes in a double nanohole with excitation polarization parallel to the apex. (**d**) Number of detected molecules in the apex region as function of the molecular concentration. The slope of the curve quantifies the apex near-field volume *V*_*eff*_.

For fluorescence experiments, the DNH structure is first cleaned by UV-ozone treatment for 10 minutes to remove organic impurities and render the gold surface hydrophilic^32^,^39^,^40^. Next, the DNH structure is covered by the solution containing the fluorescent probe molecules (Alexa Fluor 647 from Invitrogen, Carlsbad, CA) at micromolar concentrations along with 200 mM methyl viologen (1,1′-Dimethyl-4,4′-bipyridinium dichloride, Sigma-Aldrich). The use of methyl viologen quenches the dye quantum yield to 8% and maximizes the fluorescence enhancement^14^,^28^. Figure 2a shows the raw fluorescence intensity traces in a DNH with 20 *μ*M Alexa Fluor 647 and 200 mM methyl viologen. A higher fluorescence intensity is obtained when the excitation polarization is set parallel to the apex region between the double nanohole structure, in accordance with the higher excitation intensity expected from the simulations (Fig. 1c). To characterize the apex detection volume and the fluorescence enhancement, we perform fluorescence correlation spectroscopy (FCS) analysis and compute the temporal correlation of the intensity traces in (a). The FCS data supports the polarization dependency of the DNH (Fig. 2b): a high FCS correlation amplitude is found when the excitation is set parallel to the apex region, which relates to a reduced number of molecules within the nanoscale detection volume (the FCS amplitude scales inversely with the number of detected molecules, see Methods section for details). The confocal measurement for the reference solution (without nanostructure) shows comparatively high average fluorescence intensity (green curve in Fig. 2a) and very weak FCS correlation amplitude (Fig. 2b). This corresponds to the expected situation that at 20 *μ*M there are about 6200 molecules in the 0.5 fL diffraction-limited confocal detection volume with a low average brightness per molecule (the brightness per molecules in confocal setup is *Q*_*sol*_ = 0.17 kcounts/s at 10 *μ*W excitation due to the presence of the chemical quencher).

The fitting parameters for the FCS analysis are summarized in Table 1. In the case of excitation polarization parallel to the DNH apex, we obtain an average number of N^*^ = 0.9 molecules in the hot spot with brightness Q^*^ = 15.6 kcounts/s. These values correspond to a fluorescence enhancement of Q^*^/*Q*_*sol*_ = 92, and a hot spot volume of 74 zL (1 zL = 10^−21^ L), equivalent to a detection volume reduction of *N*_*sol*_/N^*^ = 6900. In addition to the fluorescence enhancement and nanoscale confinement of light, the FCS curves in DNH also show polarization-dependent microsecond residence time in the apex region, which is consistent with the 25 nm gap size and overrule the occurrence of molecular adhesion to the metal surfaces.

To demonstrate the control and reproducibility of our experiments, we conduct a series of FCS measurements with increasing concentrations of fluorescent dye (Fig. 2c). As expected, the increase in fluorophore concentration results to higher number of molecules within the detection volume and lower amplitude of the correlation curves. The linear relationship between the number of detected molecules (N^*^) in the near-field region with the fluorophore concentration confirms the effective detection volume *V*_*eff*_ of 74 zL (Fig. 2d). Remarkably, this volume corresponds very well to the geometrical dimensions of the apex region of 60 × 25 × 50 nm^3^ = 75 zL, considering a typical thickness of 50 nm for the intensity profile decaying evanescently inside the DNH obtained from numerical simulations (see Fig. 1c insert).

**Figure 3:**
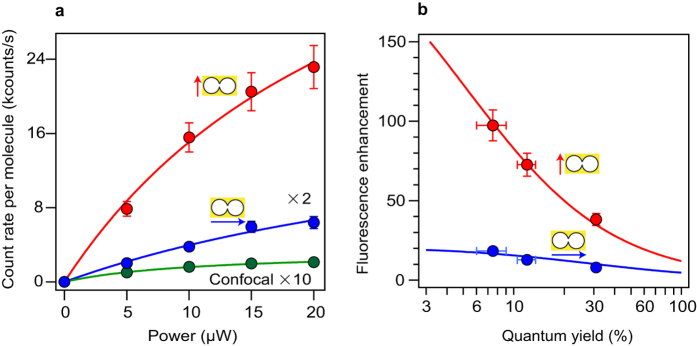
(**a**) Fluorescence brightness per molecule versus the excitation power for Alexa Fluor 647 with 200 mM methyl viologen (quantum yield ~8%). The data for the double nanohole with perpendicular orientation respective to the apex (blue) and the reference confocal data (green) are multiplied respectively by 2× and 10×. (**b**) Fluorescence enhancement factors with excitation polarization parallel (red) and perpendicular (blue) respective to the apex. Different concentrations of chemical quencher are used, corresponding to different values of quantum yield in solution: from left to right the data points correspond to methyl viologen concentrations of 200 mM, 80 mM and 0. For (**b**), the excitation power is 10 *μ*W.

In Fig. 3a we vary the excitation power and report the average fluorescence brightness per molecule for both polarization orientations. In the DNH with parallel orientation, count rates per molecule above 20 kcounts/s can be readily obtained, while for the confocal reference the fluorescence brightness saturates to values below 1 kcounts/s in the presence of methyl viologen. The experimental points follow the general model of the fluorescence brightness *AI*_*e*_/(1 + *I*_*e*_/*I*_*s*_), where *I*_*e*_ is the excitation power, *I*_*s*_ the saturation power, and *A* is a constant proportional to the molecular absorption cross-section, quantum yield, and setup collection efficiency^27^.

The effect of the fluorophore’s quantum yield is studied using different concentrations of methyl viologen. Figure 3b summarizes the fluorescence enhancement results for both DNH polarization orientations and the three cases of 200 mM, 80 mM, and no methyl viologen. The fluorescence enhancement factor increases significantly from 40 to 100× while Alexa Fluor 647 quantum yield is quenched from 30% to 8%. This behavior is well taken into account by a model of the fluorescence enhancement factor *η*_*F*_ as function of the fluorophore’s quantum yield in reference solution *ϕ*^41^,^42^:


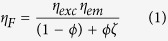


where *η*_*exc*_ is the excitation intensity enhancement, *η*_*em*_ is the radiative rate enhancement times the collection efficiency enhancement, and 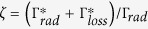 is a parameter describing the ratio of the radiative rate 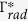 and the nonradiative rate to the metal 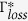 due to ohmic losses relative to the dye’s radiative rate Γ_*rad*_ in confocal reference. We will show in the next section that *ζ* is actually equivalent to the enhancement of the local density of optical states (LDOS). Therefore, in the analysis of the data in Fig. 3b using Eq. (1), we set the value of *ζ* to the LDOS enhancement found experimentally from the fluorescence decay dynamics. As shown in Fig. 3b, the agreement with the Eq. (1) model and the experimental data is excellent for both polarizations. Assuming that *η*_*exc*_ ≈ *η*_*em*_ and neglecting the gain in collection efficiency^28^, the extrapolation of the data to *ϕ* → 0 indicates a local intensity enhancement of *η*_*exc*_ ~ 14 for parallel and ~4 for perpendicular orientation, in good agreement with the numerical simulations in Fig. 1b,c.

### Fluorescence photodynamics acceleration and LDOS enhancement

**Figure 4:**
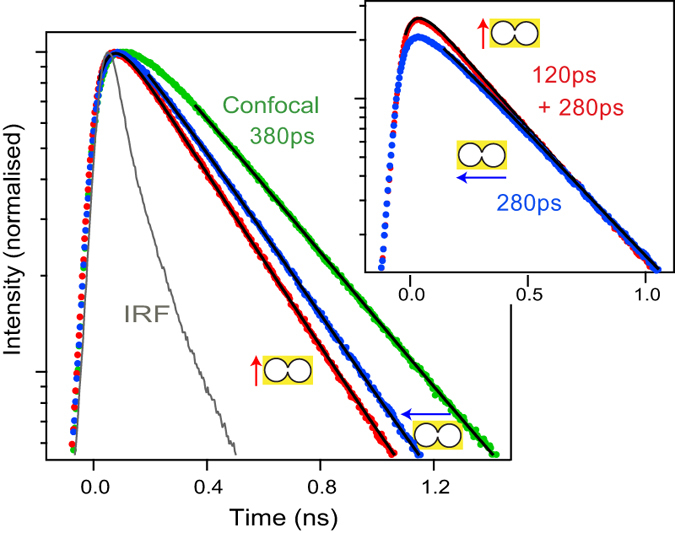
Amplitude-normalized fluorescence decay traces with excitation light parallel (red line) and perpendicular (blue line) to the apex region. The decay trace with the diffraction-limited volume (green) provides the reference for Alexa Fluor 647 with 200 mM methyl viologen. Black lines are numerical fits used to determine the fluorescence lifetime indicated on the traces. IRF denotes the instrument response function. For a supplementary comparison between parallel and perpendicular cases, the inset displays the traces normalized so that the longer time decay component has a similar amplitude for both cases. The additional short lifetime contribution representative of the apex region clearly emerges when the polarization orientation is parallel to the apex.

Time correlated single photon counting (TCSPC) measurements record the fluorescence decay kinetics upon picosecond pulsed excitation. Figure 4 displays typical decay traces for the confocal reference and the DNH with excitation polarization parallel and perpendicular to the apex. The TCSPC data show clear acceleration of the decay dynamics from confocal to DNH and from perpendicular to parallel orientation. Fitting the TCSPC data with a single exponential model (black lines in Fig. 4) provides the fluorescence lifetime for the confocal case and DNH with perpendicular orientation. For the DNH with parallel orientation, we use a bi-exponential model to account for the respective contributions of the N^*^ molecules in the apex region and the *N*_0_ molecules in the nanoholes (outside the gap). For each case, the model takes into account the temporal resolution of our apparatus by computing the (re)convolution of the exponential decay with the instrument response function (IRF, full width at half maximum 120 ps)^27^,^41^.

In the presence of 200 mM methyl viologen, the Alexa Fluor 647 fluorescence lifetime becomes 380 ps. For the DNH with perpendicular orientation, the presence of the nanohole further reduces this lifetime to 280 ps (1.35× lifetime reduction), a value that is similar to the lifetime reduction obtained with single gold apertures^27^. For the DNH with parallel orientation, two exponential decays are observed (see insert in Fig. 4): a fast 120 ps decay corresponding to the N^*^ molecules in the gap, and a longer 280 ps decay for the *N*_0_ molecules outside the gap. Remarkably, the longer decay time corresponds to the decay time obtained for perpendicular orientation, confirming that the background fluorescence stems mainly from the nanohole region.

With the use of 200 mM methyl viologen, the chemical quenching rate Γ_*q*_ represents a large fraction of the total decay rate Γ_*tot*_ (inverse of fluorescence lifetime). To estimate the LDOS (local density of optical states) enhancement with the DNH, the influence of the chemical quenching rate Γ_*q*_ must be taken into account prior to computing the ratio of decay rates. The LDOS encompasses both radiative and non-radiative transitions set by the photonic environment (such as energy transfer to the free electrons in the metal)^43^. However, the LDOS is not proportional to the chemical quenching rate set by the presence of methyl viologen^44^. To estimate the amount of decay rate that actually depend on the LDOS, we write the dye total decay rate in the confocal case as Γ_*tot*_ = Γ_*rad*_ + Γ_*nr*_ + Γ_*q*_, where Γ_*rad*_ denotes the radiative rate, and Γ_*nr*_ is the internal non-radiative decay rate. In the presence of the DNH, the decay rate becomes 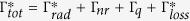. We have added a supplementary term 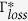 to account for non-radiative energy transfer to the metal, and we assume that the internal non-radiative decay rate Γ_*nr*_ and the methyl viologen quenching rate Γ_*q*_ are independent of the presence of the DNH^44^. The LDOS enhancement is then obtained as 
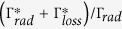
, keeping only the rate influenced by the photonic environment, and taking into account non-radiative transfer to the metal. This expression of the LDOS enhancement corresponds to the quantity *ζ* used in Eq. (1).

We estimate the internal non-radiative rate Γ_*nr*_ = 0.67 ns^−1^ and the quenching rate Γ_*q*_ = 1.75 ns^−1^ using the knowledge of the 30% quantum yield of Alexa Fluor 647 in pure water solution which is quenched to 8% by 200 mM methyl viologen (a detailed Stern-Volmer analysis is presented in the supporting information of^28^). We can now subtract these values of Γ_*nr*_ and Γ_*q*_ from the total decay rate 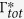 with the DNH so as to estimate the part depending on the LDOS. For the DNH with perpendicular orientation, we get 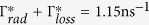 and a LDOS enhancement of 5.5× which is characteristic of nanoholes^27^. For the parallel orientation, the apex further influences the decay rates so that 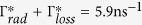. This corresponds to a LDOS enhancement of 5.9/0.21 = 28×, providing a clear demonstration of the DNH apex significant influence on the LDOS. Table 2 summarizes the different rates, providing a complete overview of the fluorescence photokinetics alteration in the DNH. For the enhancement of the radiative rate Γ_*rad*_, we use the value of *η*_*em*_ deduced from Fig. 3b. The analysis of Table 2 also reveals the increase of the non-radiative losses Γ_*loss*_ to the metal, which contribute to quench the fluorescence emission. Fortunately, this electromagnetic quenching is compensated by the simultaneous increase in radiative rate Γ_*rad*_ (Purcell effect), so that the effective quantum yield *ϕ* of the dye is actually increased by the DNH presence.

**Figure 5:**
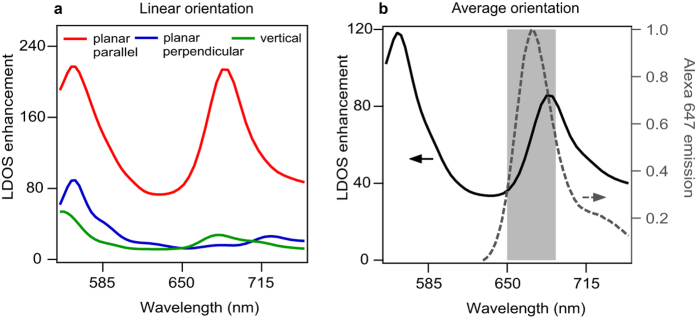
Numerical simulations of LDOS enhancement for a dipolar emitter located in the center of the DNH gap. In (**a**), three different dipole orientations are displayed, the case when the dipole is oriented parallel to the apex provides the highest LDOS enhancement. In (**b**), the orientation-averaged LDOS enhancement is plotted as function of the emission wavelength (solid line). The normalized Alexa Fluor 647 emission spectrum is shown in dashed gray line, and the 650–690 nm region used experimentally for fluorescence collection is indicated.

To corroborate the experimental findings, we compute the LDOS enhancement as the relative increase in power released by a dipolar emitter located at the center between the DNH apex^42^,^45^. Figure 5a shows the LDOS enhancement for a dipole with different orientations. The case of orientation parallel to the DNH apex clearly stands out with a maximum LDOS enhancement up to 210×. Considering the orientation-averaged LDOS enhancement in the 650–690 nm region (Fig. 5b), we obtain a mean 60× LDOS enhancement. This value is within a factor 2 of the experimental observation, which is satisfactory considering the 3D spatial averaging in the experiments, the limited temporal resolution of our apparatus, and some minor nanofabrication deficiencies. Moreover, the simulations show that the DNH design bears a resonance around 685 nm that covers well the Alexa Fluor 647 emission spectrum.

## Conclusion

We provide a complete picture of the enhanced emission from nanoscale DNH volumes by analyzing the fluorescence temporal dynamics from pico- to milliseconds time scales. The clear polarization-dependent response allows to extract the relevant signal from the 25 nm gap area. We measure a nanometer detection volume of 74 zL, 7000-fold below the diffraction-limited confocal volume, and report fluorescence enhancement up to 100-fold, together with local density of optical states (LDOS) enhancement around 30-fold. As compared to state-of-the-art antenna-in-box design, the DNH has a comparatively lower detection volumes due to a better lateral and axial confinement. It is also significantly easier to fabricate using simple focused ion beam patterning. The DNH spectral resonance occurs in the range 550–700 nm, while it is in the near-infrared for the antenna-in-box. The DNH therefore enables a better spectral overlap with the emission band of most common red fluorescent dyes. While the antenna-in-box provides a higher gap intensity thanks to a nanoantenna disconnected from the metal film, the DNH partly compensates this feature by lower non-radiative losses and a better spectral overlap with the resonance. We also point out that much room remains for a thorough optimization of the DNH design parameters. Altogether, the distinctive features of double nanoholes combining high enhancement, efficient background screening and relative nanofabrication simplicity, open promising perspectives to study complex biochemical dynamics at physiological concentrations.

## Methods

### Double nanohole fabrication

The double nanohole structure is milled using focused ion beam (FEI Strata DB235) in a 100 nm thick gold film adhered to the glass substrate with 5 nm Ti adhesion layer. Prior to all experiments, the sample is cleaned by UV-ozone treatment for 10 minutes to remove organic impurities and render the gold surface hydrophilic. Experiments are performed immediately afterwards, with the sample being exposed to air for less than 2 minutes. After the FCS experiments, the sample is rinsed with ethanol, dried with nitrogen and cleaned again under UV illumination for 10 minutes. With this protocol, the sample can be reused several times without observing any change in optical performance (see the experiment series in Fig. 2).

### Experimental setup

The fluorescence experiments are carried on a confocal inverted microscope (40×, 1.2 NA water-immersion objective) equipped with a three-axis piezoelectric stage for precise positioning of nanostructure within the laser focus. The excitation source is a linearly polarized He-Ne laser at 633 nm with 10 *μ*W incident on the sample. Out-of-focus fluorescence is rejected by a 30 *μ*m pinhole conjugated to the sample plane. Finally, the fluorescence is recorded using two avalanche photodiodes with 670 ± 20 nm bandpass filters. Further for time-correlated counting measurements, the excitation source is switched to a picosecond pulsed laser diode operating at 636 nm (PicoQuant LDH-P-635, with 80 MHz repetition rate).

### Fluorescence correlation spectroscopy method to quantify the apex volume and fluorescence enhancement

To quantify the hot spot detection volume and fluorescence enhancement, we analyze the fluorescence intensity temporal fluctuations *F*(*t*) with a hardware correlator (Flex02-12D/C correlator.com, Bridgewater NJ, 12.5 ns minimum channel width) to perform fluorescence correlation spectroscopy (FCS). FCS computes the temporal correlation of the fluorescence signal 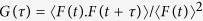, where *τ* is the delay (lag) time, and 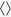 indicates time averaging^46^,^47^.

In the DNH, the total fluorescence signal is the sum of the enhanced fluorescence from molecules within the apex region and the fluorescence from the molecules present in each nanohole. The FCS analysis discriminates between these contributions by considering the trace as a sum of two molecular species with different number of molecules and brightness: N^*^ molecules within the apex region with brightness Q^*^, and *N*_0_ background molecules with brightness *Q*_0_ diffusing away from the region of interest (essentially inside the two nanoholes). An essential feature in FCS is that the molecules contribute to *G* in proportion to the square of their fluorescence brightness^47^, so that the fluorescence from molecules in the apex region experiencing the maximum enhancement will have a major contribution in the FCS correlation^28^,^48^. The temporal correlation of the fluorescence intensity *F* can be written as^47^:


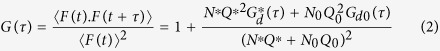


where 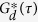 and 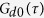 are the normalized correlation functions for each species taken individually based on a classical three dimensional model:


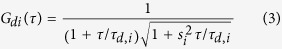


*τ*_*d,i*_ stands for the mean residence time (set by translational diffusion) and *s*_*i*_ is the ratio of transversal to axial dimensions of the analysis volume, whose value is set to *s* = 0.2 as it has negligible influence on the estimates of molecular concentration and brightness within the apex region (N^*^, Q^*^).

To extract the number of molecules within the apex region (N^*^) and the corresponding fluorescence brightness Q^*^ (for a given sample concentration), we use the asymptotic value of the correlation function towards zero lag time^28^:


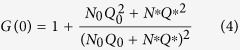


The value of total fluorescence intensity *F* (i.e, *N*_0_*Q*_0_ + N^*^Q^*^) is known from the experimental measurement, thus replacing N^*^Q^*^ = *F* − *N*_0_*Q*_0_ into Eq. (4), we obtain the fluorescence brightness and number of molecules within the apex region:


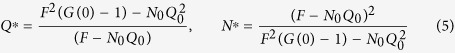


These expressions show that in addition to the experimentally measured parameters *F* and *G*(0), we need to estimate the number of molecules and brightness (*N*_0_, *Q*_0_) for the molecules diffusing away from the apex region. At 20 *μ*M concentration, we get *N*_0_ = 49, and *Q*_0_ = 1.95 kcounts/s from the results obtained from a control experiment with a double nanohole without any connecting gap region. These experimental findings are further validated from earlier work on single nanoaperture^27^,^41^.

### Numerical simulations

We performed two sets of finite-difference time-domain (FDTD) simulation (Lumerical 8.1). First, transmission through the DNH aperture was correlated with the experimental results. The SEM image of the structure was imported to the simulation software environment. We used a total-field scattered-field source and the 3D simulation region was enclosed with perfectly-matched-layer boundaries with a 2 nm mesh override (1 nm mesh for the intensity maps in Fig. 1b,c). The permittivity of gold was taken from Johnson and Christy^49^ and the refractive indices for the glass substrate and water were set to be 1.52 and 1.33. Second, the LDOS enhancement was calculated where dipole emitter located at the center between the cusps of the DNH was used to mimic the emission of the fluorescent molecule^42^,^45^. The imaginary part of the Green’s Function was evaluated along three axes, i.e. Imag(*G*_*xx*_), Imag(*G*_*yy*_), Imag(*G*_*zz*_); and from that partial LDOS along the three axes were obtained and then averaged. The averaged LDOS was normalised to that of a homogeneous water medium.

## Additional Information

**How to cite this article**: Regmi, R. *et al.* Nanoscale volume confinement and fluorescence enhancement with double nanohole aperture. *Sci. Rep.* **5**, 15852; doi: 10.1038/srep15852 (2015).

## Figures and Tables

**Table 1:** Fitting parameter results for the FCS curves obtained on double nanohole (Fig. 2b).

Excitation polarization	Confocal	Double nanohole	
	Linear	Parallel	Perpendicular
*F* (kcounts/s)	1090	110	85
G(0)-1	0.16 × 10^−3^	34 × 10^−3^	22 × 10^−3^
*N*	6200	0.9	46
*τ*_*d*_(*μ*s)	62	1.2	33
*Q* (kcounts/s)	0.17	15.6	1.9
Detection volume (zL)	500 × 10^3^	74	4 × 10^3^
Fluorescence enhancement		92	11
Volume reduction		6900	140

The polarization orientation is respective to the DNH apex. For the DNH-parallel case, the FCS fit considers two species. The number of molecules and diffusion time for the slowly diffusing species (aperture region) are respectively *N*_0_ = 49 and *τ*_*d,*0_ = 33 *μ*s (see Methods section for details).

**Table 2:** Fluorescence photokinetic rates inside DNH: Γ_*rad*_ radiative rate, Γ_*loss*_ non-radiative transitions to the metal, Γ_*nr*_ intramolecular non-radiative transitions, Γ_*q*_ methyl viologen quenching rate, Γ_*tot*_ total decay rate (inverse of fluorescence lifetime), *ϕ* quantum yield.

	Γ_*rad*_	Γ_*loss*_	Γ_*nr*_	Γ_*q*_	Γ_*tot*_	*ϕ*
Confocal	0.21	–	0.67	1.75	2.63	0.08
DNH perpendicular	0.84	0.31	0.67	1.75	3.57	0.24
DNH parallel	2.94	2.97	0.67	1.75	8.33	0.35

All rates are expressed in ns^−1^, the typical uncertainty is ±0.05 ns^−1^.
